# On the Exploration of Adaptive Mechanisms Providing Reliability in Clustered WSNs for Power Plant Monitoring

**DOI:** 10.1155/2016/4158735

**Published:** 2016-01-17

**Authors:** Sathiyaseelan Rathinavel, Vijayakumar Pandi, Audithan Sivaraman

**Affiliations:** ^1^Department of Computer Science and Engineering, Anna University, Chennai, Tamil Nadu 600025, India; ^2^Department of Computer Science, University College of Engineering, Tindivanam, Tamil Nadu 604001, India; ^3^Department of Electronics and Communication Engineering, PRIST University, Thanjavur, Tamil Nadu 613403, India

## Abstract

Wireless Sensor Networks (WSNs) are used in almost every sensing and detection environment instead of wired devices in the current world, all the more in power plant monitoring applications. In such a kind of environment, providing reliability is a challenging task, since WSN makes use of low powered sensors. There are many existing works that provide reliable transmission in WSN (predominantly via multipath routing). However, most of the existing works take additional delay, excessive packet loss, and energy consumption, and hence they provide less packet delivery and throughput. Adaptive Priority Routing (APR) is first proposed during the initial design to provide efficiency in next hop selection. APR computes the priority value for selecting the intermediate nodes during the data transmission in order to improve the packet delivery, throughput, and energy efficiency. In addition to this, APR is developed into QAPR protocol to provide reliability which can operate in two modes, *D* representing distance mode and *Q* representing quality of service (QoS) mode. The proposed work is simulated in both flat topology and hierarchical topologies and the simulation analysis shows that the reliability is increased significantly in comparison with existing works.

## 1. Introduction

Wireless Sensor Network (WSN) can be defined as an assemblage of discrete sensors in order to oversee and account for the substantial states at the deployed expanse to a central locality (base station, BS). Although the first application to deploy WSNs was to monitor country borders, eventually they are used to check physical wellbeing, traffic control, and many other domestic and commercial domains. An antenna, favorably an Omniantenna, is interfaced using an electronic circuit. A processor or a controller unit is used to manipulate the data across the node, usually powered by any battery source. The cooperative operation of the various modules builds a node, and the nodes build a WSN.

WSN can be viewed as a network which collects the information from its surrounding environment and sends its sensed data to the BS. When the word “routing” is used, it generally means the course of action of selecting the best paths in the WSN between various nodes for data transmission. Routing of the incoming data in WSNs is executed at the third layer of the OSI model (the network layer). A multihop WSN generally possesses the intermediate sensing nodes that may require relaying their packets towards base station. In such kind of situation, the routing protocols designed for WSNs must accomplish high reliability. There have to be manifold paths from source node to destination node relaying data in order to accomplish robustness. The faulty performance of even a single node may lead to unexpected changes and/or degraded network operation. This kind of action can be a consequence of energy drain which highlights the significance of energy management in a WSN. WSNs applications are not limited to measure parameters concerning surrounding circumstances such as heat level, altitude, pressure, wind speed, intensity of contamination, moisture level in the atmosphere, spectral variation, and movement.

**Figure 1:**
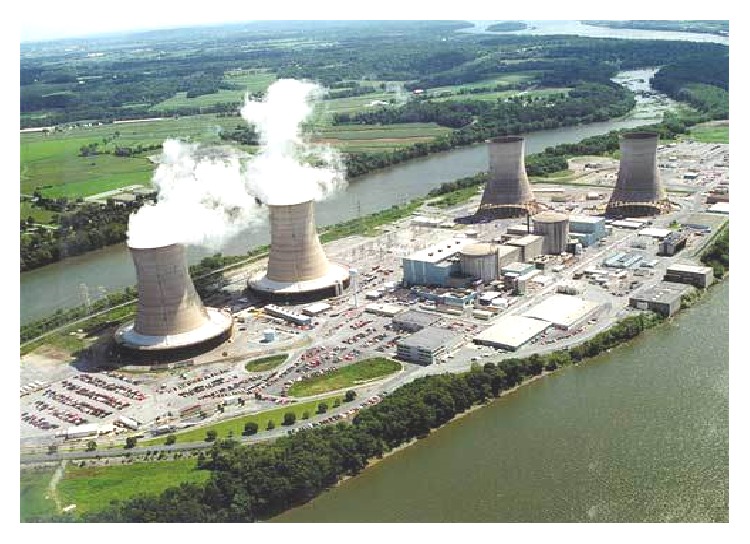
An earth view of the MAPS, Chennai, India.

Many types of sensors can be found in power plant monitoring systems like flow sensors, radiation sensors, pH sensors, chemical sensors, RPM sensors, level sensors, biosensors, temperature sensors, pressure sensors, voltage sensors, vibration sensors, and so forth. There are two types of messages that could be possibly generated. The first one is a regular update *M*_*U*_ to the controlling unit and the second one is an alert message *M*_*A*_ caused by any abnormal or unpredicted observation of such parameters. Generally, an alert message is of great priority indicating a mishap in the hostile area, in this case, for detection of power failures, a fire accident, or any unexpected occurrence in the atomic power station. The QAPR protocol proposed in this paper has been mainly designed for the Madras Atomic Power Station (MAPS), India (shown in Figure 1).

**Figure 2:**
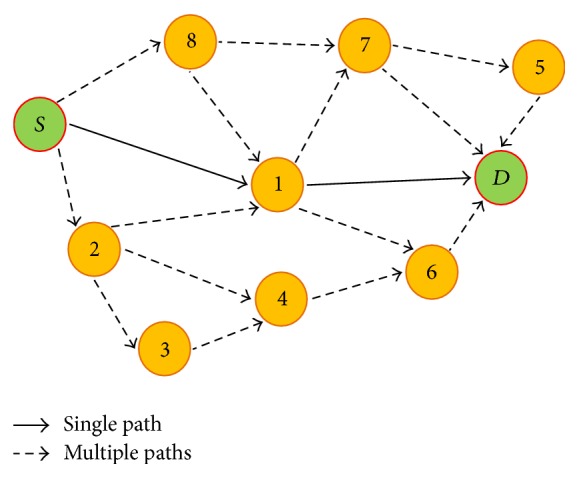
Flat topology.

Data transmission has been considered as one of the most common schemes for improving transmission reliability in WSN [1]. Acknowledgment/negative acknowledgement (ACK/NACK) messages are the basic method used to access the necessity of retransmission. Nevertheless, such method generates extra traffic causing additional overhead that makes it less applicable in highly constrained and error prone environments, like WSNs. Generally, multipath routing is preferred to improve reliability in WSNs [2]. However, multipath routing causes a very high energy consumption rate. To understand this, Figure 2 shows multipath communication from source (*S*) to destination (*D*).

A single path from the source to destination involves single hop transmission in Figure 2. Therefore, the number of transceptions performed is at least 2(*n* + 1), whereas in multipath transmission, the number of transceptions performed is at least 2(*n* + 1) + *kn*, where *k* is the number of intermediate nodes relaying data in multipath communication. Therefore, performing multipath communication merely to improve reliability is an energy consuming process. Proactive mechanisms like EARQ [3] use a route update mechanism to keep the reliable route updated for every communication operation and this induces additional overhead in the network. Thus, it becomes a difficult choice between proactive or reactive routing mechanisms for an atomic power plant application. Most reactive routing mechanisms provide reliability since they work on a feedback basis and proactive routing induces excessive overhead into the WSN. This issue of providing reliability in a reactive manner for WSNs to monitor an atomic power plant with low energy consumption and high quality of service is the main focus of this paper.

The major research impartations of this paper are as follows:

(i) The design of an Adaptive Priority Routing (APR) protocol based on average QoS, average energy, and average interference in the neighbourhood to improve per-hop packet delivery is studied.
(ii) Having identified a limitation of “Cart before the Horse” issue in the APR mechanism, Quality Adaptive Priority Routing (QAPR) can minimize the effect of local judgment, thereby reducing delays while operating in the two modes *D* and *Q*.
(iii) QAPR improves energy efficiency and reliability without the need for multipath routing for clustered networks using single hop reliability estimation.

The remainder of this paper is organized as follows.  Section 2 analyzes the related works.  Section 3 presents the system models under consideration.  Section 4 describes the need for Adaptive Priority Routing (APR) to improve per-hop delivery in WSNs.  Section 5 introduces QAPR protocol to improve reliability in clustered multihop WSNs. An account of the simulation results along with the comparative performance analysis of the proposed system is given in Section 6. Lastly, the conclusion and future work are found in Section 7.

## 2. Related Works

Many works that have correlated with the work proposed in this paper are available in the literature. They are classified as clustering and routing protocols based on the adaptive nature in the WSN.

### 2.1. Clustering Protocols

One of the earliest adaptive protocols for communication in WSN is Low Energy Adaptive Clustering Hierarchy (LEACH) [4]. By forming cluster, the energy usage is low within the cluster but drains the energy resource for the deterministic cluster head (CH) selected. The incorporation of adaptivity in the network is achieved by means of the CH selection. One of the main problems which LEACH protocol consists of is the additional energy expenditure of the CH in comparison to other nodes. Energy-efficient routing was performed in WSNs through balanced clustering [5] protocol. The Gaussian elimination algorithm is applied to minimize energy utilization per round. The authors remodelled the energy transmission equation to suit the scenario considered. The major advantage of the ECHERP protocol is the achievement of balanced clustering and routing through the selection of energy optimal node unlike traditional protocols that choose high node. The lack of investigations on whether the QoS metric requirements are met is the major drawback of this work.

Distributed Weight-Based Energy-Efficient Hierarchical Clustering (DWEHC) protocol for WSNs [6] generates well balanced clusters. This method employs the hierarchical level of a node depending on two important facts: the range of a cluster and the path to the CH with minimum energy requirement. The advantage of the two-stage technique is that it exhibits huge progress in both intra- and intercluster energy consumption. The clustering procedure is absolutely independent of the size of the network.

The Unequal Clustered Routing (UCR) algorithm [7] proposed an Energy-Efficient Unequal Clustering (EEUC) mechanism. The region based clustering is performed to divide the nodes into unequal clusters. This is achieved by picking any node randomly and checking whether it lies within the competitive radius or not. The expression for checking this criterion is given in

(1) $$\begin{array}{c} R_{i}=\left(\;1-c\frac{d_{\max}-d\left(s_{i},\text{BS}\right)}{d_{\max}-d_{\min}}\;\right)R_{0}, \end{array}$$

where *d*_max⁡_ is the maximum distance of the nodes from BS {0 ≤ *d*_max⁡_ < *∞*}, *d*_min⁡_ is the minimum distance of the nodes from BS {0 ≤ *d*_min⁡_ < *∞*}, *d*(*s*_*i*_, BS) is the distance of the tentative head *s*_*i*_ from BS {0 ≤ *d*(*s*_*i*_, BS) < *∞*}, and *R*_0_ is the maximum competition rate {0 ≤ *R*_0_ < *∞*}.

This model outperforms Hybrid, Energy-Efficient, Distributed Clustering (HEED) protocol [8] in the energy consumption especially. Many other energy-efficient clustering algorithms are available [9]; however, they lack various aspects of quality of service while merely improving energy efficiency.

### 2.2. Routing Protocols

In Ad hoc On-demand Distance Vector (AODV) routing [10], route construction is an uncomplicated process, where the broadcasting of route request (RREQ) by the source is the first sign of route discovery. Two sequence numbers (sqns) are seen in the RREQ: one is for the source and another for the destination. The source sqn is used to check the liveliness of the RREQ and destination sqn is to ensure the liveliness of the route that can be established by the destination. In response to a unique RREQ message, a route reply (RREP) is produced to show the route for data to traverse. The adaptive nature of this protocol is exhibited while routing information across the nodes by dynamic generation of routes.

Destination Sequenced Distance Vector (DSDV) [11] adapts routes and sqns of the Bellman Ford Algorithm dynamically at each node into a table, to help in attaining the shortest routes to destination in a proactive manner. This algorithm aims to solve the root loop problem. It needs a regular update of its routing table, a process by which battery power is rapidly consumed and a minute part of bandwidth is used up even under idle condition. Dynamic Source Routing (DSR) [12], another technique that performs source routing reactively like AODV, uses the cache memory for trouble-free route formation. The necessity to flood the network using table update messages at fixed intervals is eliminated by the reactive approach of DSR. Although the protocol performs well enough in stationary condition and conditions with low mobility of nodes, the increase in mobility causes increase in performance degradation.

The Energy Aware Routing (EAR) protocol [13] tries to warrant the surviving ability of networks with meagre energy levels. Similar to AODV, this method is reactive as well and it exhibits directed diffusion. A unique optimal route that could be used for communication is not determined by EAR, which is a disadvantage observed. The disadvantage is observed here. Instead it keeps a set of good paths and probabilistically picks a path. EARQ is another reliability based protocol that assures the routing efficiency by estimating a route based on the energy expenditure and cost to route through each successive node [3].

The measurement analysis provides a realistic reliable route to the destinations during every communication performed, in spite of the probabilistic route selection nature of the protocol. The drawback of this protocol, however, is that the previous state of this node in terms of quality of service or redundancy of communication is not considered dynamically. EDEAR [14] improves two significant conditions, energy consumption and end-to-end delay, dynamically. Unlike general routing protocols, the BS here discovers alternate paths for communication. A probability method picks the path according to a multicriteria cost function which allows minimizing both the network delay and the energy consumption. There are many advantages for this protocol over other protocols, except for the fact that it does not take the Signal-to-Noise Ratio (SNR) into consideration. Received Signal Strength (RSSI) is utilized for SNR calculation.

Adaptive Packet Scheduling (APS) scheme was proposed [15] to support real-time traffic in WLAN Mesh Networks where the performance is improved by assuring inter- and intraclass service demarcation of the packet. APS establishes a way to fairly allocate resources within the service classes. The mechanism clearly assigns priority for a packet after dividing into four major classes. The APS scheme improves QoS for real-time traffic in packet reception and fairness.

Adaptive reliable routing based on cluster hierarchy for wireless multimedia sensor networks [16] includes energy prediction and power allocation mechanism. Two key methods that were brought forth include energy forecast and power assignment. This routing attempts to bring equilibrium among nodes in terms of energy usage. For achieving superior network operation standards, the clusters are formed by cellular topologies. The node transmission power is adjusted by an allocation mechanism designed and to further incorporate energy awareness in the same.

Adaptive reliable routing protocol was proposed for WSNs [17], where the overhearing feature characterizes the wireless channels as an implied acknowledgement system. Besides this, adaptive selection of path is established using accumulative coordination inside the region. This strategy appears to be effortless and proficient; however, it does not provide maximum packet delivery. Energy-efficient adaptive multipath routing [18] intends to provide a consistent transmission network at minimal energy utilization. This is achieved by the economic use of the power accessibility and the RSSI to recognize numerous paths. The major limitation in this scheme is that there are chances for variable RSSI values recorded during simulation, which make it unsuitable for mapping with the real world. Additionally, the fading and interference occurring in the WSN atmosphere are ignored. Adaptive majority based rerouting was proposed for differentiated delay [19]. The method focuses on establishing a model with delay called MRHD. This factor is held responsible for bringing about quality in routing. As the route is established, there is classification of data into various classes observed. Furthermore, MRHD utilizes an adaptive strategy to send data through paths that cause less delay.

Priority based routing for solar powered WSNs was proposed to improve reliability [20]. The APOLLO algorithm estimates KR metric at regular intervals after obtaining local information pertaining to energy model of a solar cell, so as to exploit complete energy. Taghikhaki et al. [21] proposed REC+ routing to provide reliability in clustered multihop networks. This technique uses a d-hop reliability technique to provide reliability in the routing of WSNs, which is contrary to the system proposed in this paper. QoS requirements are also met by this protocol using a few assumptions.

Compared to the existing approaches available in the literature, the work presented in this paper is different in many aspects. The proposed work firstly maximizes the packet delivery ratio and throughput in the WSN. Secondly, the work proposed here minimizes the packet loss ratio and delay occurring in the WSN. Energy efficiency is appreciably improved in the mechanisms proposed with the two system models considered.

## 3. System Model

### 3.1. Flat Topology

**Figure 3:**
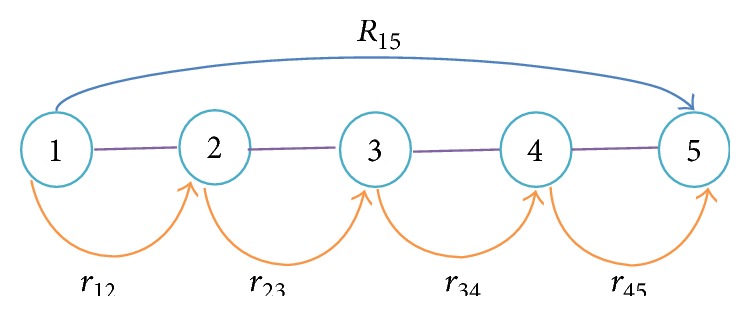
Flat topology.

Two models are simulated in this paper; first a flat topology is considered to identify the problem and define the system model. Reliability is lacking in most routing protocols. Considering Figure 3, node 1 is the source to transmit data to destination 5. The reliability to send data end to end (*R*_15_) remains much lesser than the reliability from node 1 to node 2 (*r*_12_). This is identified hence and every set of neighbours of the current source is assessed for the best next hop to forward data reliably to the destination.

Assume that there is one multihop path. The reliability between nodes *i* and *j* is *r*_*ij*_, and the reliability from node 1 to node 5 is *R* or *R*_*ij*_. The communication should convene the criterion as given in

(2) rij≥Rij,1≤i≤4,  i+1=j,∏i=1,j=i+1i=n−1,j=nrij≥R,

where *n* describes the total number of nodes. It is quite sensible and reasonable to estimate reliability hopwise and select node based on that factor.

### 3.2. Hierarchical Topology

**Figure 4:**
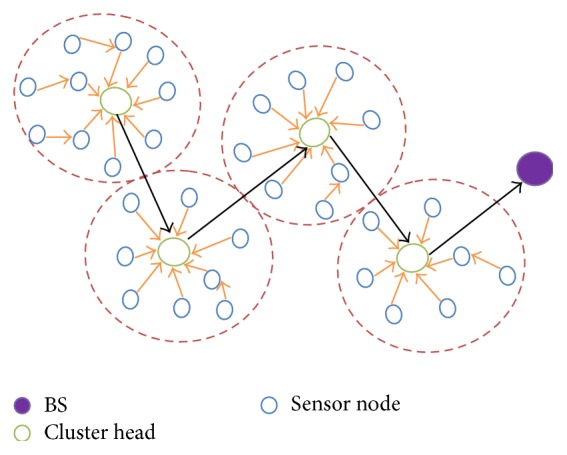
Hierarchical topology.

Secondly, we adopt a WSN formed by *n* random deployed sensor nodes, *m* cluster heads, and BS. Similar to flat topology, all nodes remain static in the area of deployment. The cluster heads collect sensed parameters in each group and report to the BS.  Figure 4 shows the arrangement of the nodes in the hierarchical topology. The cluster head will be responsible for sensor coordination relying on the QoS necessity, in conjunction with energy residual at every node. Initial energies, sensing, computation, and communication abilities are alike for all nodes. However, the base station has adequate energy as well as capacity. Reliable routing strategy is a prerequisite to send data from cluster heads to the BS. To solve these problems, two protocols are discussed in this paper: Adaptive Priority Routing (APR), which is further developed into Quality Adaptive Priority Routing (QAPR) operating in two modes: based on distance (QAPR-*D*) and based on QoS (QAPR-*Q*).

## 4. Adaptive Model Priority Routing Mechanism

In this section, we put forward Adaptive Priority Routing (APR) based on clustering for WSN. The design objective of WSN is to meet the source-destination reliability criteria. The priority selection parameter denoted by $\overset{-}{\gamma }$ for APR is derived from the general requirements for routing process: quality of service, energy conservation, and low interference communication. The assumptions made for the design of APR are listed below:

- For each source to destination communication, there will be a number of individual multihop communications.
- Each node has a list of neighbours from which the next hop will be selected.
- The requirement in each neighbour list must be satisfied by the type of communication from the current hop to the next hop.

Hence, each hop-to-hop communication is taken as the main concern for the proposed APR scheme. However, the next hop should satisfy the condition of reliable delivery using a threshold value comparison.

### 4.1. Derivation of the Priority Selection Parameter

Factors $\overset{-}{\gamma }$ and $\hat{\gamma }$ assist in picking the most reliable next node to improve the overall performance of the network operation. To estimate the value of $\overset{-}{\gamma }$ and $\hat{\gamma }$, the following values are first estimated for the set of neighbour lists (NL) of every node, each containing *L* elements (*n* ∈ NL).

*(i) Reliable Delivery Factor of a Node n*. A new reliable delivery factor for a node *n* given by $\overset{-}{\gamma }(n)$ in (3) is used for estimating the per-hop reliability of the data to the next hop. It is defined as the individual sum of SNR(*n*), *E*(*n*), and PDR(*n*), whose values are normalized and directly indicate the probability of the node in successfully delivering a packet. Hence,

(3) $$\begin{array}{c} \overset{-}{\gamma }\left(\;n\;\right)=\frac{\left(\text{SNR}\left(n\right)+E\left(n\right)+\text{PDR}\left(n\right)\right)}{3}. \end{array}$$

*(ii) Average QoS of All Neighbours*. The average QoS (QoS_avg_) is calculated based on the packet processing rate and the delay caused by a node for all the nodes present in the neighbour list. It is estimated by (4) indicating the delay:

(4) $$\begin{array}{c} {\text{QoS}}_{\text{avg}}=\frac{1}{L}\overset{L}{\underset{0}{\sum }}\frac{{\text{Pk}}_{\text{in}}\left(n\right)\times \text{Dly}\left(n\right)}{{\text{Pk}}_{\text{out}}\left(n\right)}. \end{array}$$

*(iii) Average Energy of All Neighbours*. The average energy (*E*_avg_) indicates the average of all the neighbour nodes' current energy value as in

(5) $$\begin{array}{c} E_{\text{avg}}=\frac{\sum_{0}^{L}E\left(n\right)}{L}. \end{array}$$

*(iv) Interference in the Neighbourhood*. The average Signal-to-Noise Ratio (SNR_avg_) is calculated in (6) to find whether there is too much of interference in the neighbourhood:

(6) $$\begin{array}{c} {\text{SNR}}_{\text{avg}}=\frac{\sum_{0}^{L}\text{SNR}\left(n\right)}{L}. \end{array}$$

In (3), (4), (5), and (6), the variables notations are as follows: Pk_in_(*n*) is the number of incoming packets at the node *n*, {1 ≤ Pk_in_(*n*) < *∞*}, Pk_out_(*n*) is the number of incoming packets at the node *n*, {1 ≤ Pk_out_(*n*) < *∞*}, *E*(*n*) is the current residual energy value of the node *n*, {0 < *E*(*n*) < 1}, SNR(*n*) is the Signal-to-Noise Ratio value of the node *n*, {0 < SNR(*n*) < 1}, Dly(*n*) is the normalized delay caused by the node *n*, {0 ≤ Dly(*n*) ≤ 1}, and *L* is the number of elements in the neighbourhood, {1 ≤ *L* < *∞*}. Hence,

(7) $$\begin{array}{c} \hat{\gamma }=\frac{1}{3}\left(\;\gamma_{q}+\gamma_{s}+\gamma_{e}\;\right),\;\left\{0\leq \hat{\gamma }\leq 1\right\}, \end{array}$$

where

(8) γq=QoSavgQoSmax⁡,0<QoSavg,QoSmax⁡<1,

(9) γe=EavgEin,0<Eavg,Ein≤1,

(10) γs=SNRavgSNRmax⁡,0<SNRavg,SNRavg<1.

In (8), (9), and (10), QoS_max_, *E*_in_, and SNR_max_ refer to maximum possible QOS, initial energy, and the maximum possible SNR values, respectively. Hence, to estimate the type of routing, the values $\gamma_{q}/\hat{\gamma }$, $\gamma_{e}/\hat{\gamma }$, and $\gamma_{s}/\hat{\gamma }$ are estimated. The decision on the type of routing to be performed by the current hop is decided by the greatest of the three values for data transmission.

**Case 1 .**

If $\gamma_{q}/\hat{\gamma }>\gamma_{e}/\hat{\gamma }$  and  $\gamma_{s}/\hat{\gamma }$, the node *n* with the maximum QoS belonging to the neighbour list, with the condition that $\overset{-}{\gamma }(n)$> ${\overset{-}{\gamma }}_{TH}$, is conveniently chosen as the next hop.

**Case 2 .**

If $\gamma_{e}/\hat{\gamma }>\gamma_{q}/\hat{\gamma }$ and $\gamma_{s}/\hat{\gamma }$, the node *n* with the highest energy belonging to the neighbour list, with the condition that $\overset{-}{\gamma }(n)>{\overset{-}{\gamma }}_{TH}$, is conveniently chosen as the next hop.

**Case 3 .**

If $\gamma_{s}/\hat{\gamma }>\gamma_{q}/\hat{\gamma }$ and $\gamma_{e}/\hat{\gamma }$, the node *n* with the highest SNR belonging to the neighbour list, with the condition that $\overset{-}{\gamma }(n)>{\overset{-}{\gamma }}_{TH}$, is conveniently chosen as the next hop.

**Case 4 .**

If the average energy (*E*_avg_) falls below the threshold energy level (*E*_TH_), then a pilot message is broadcasted by the next hop estimating node to indicate that it begins the highest energy routing only until the manual termination of the network operation (because atomic power plants cannot afford to operate until the entire network self-terminates operation due to energy drain).

### 4.2. Routing Strategy

**Figure 5:**
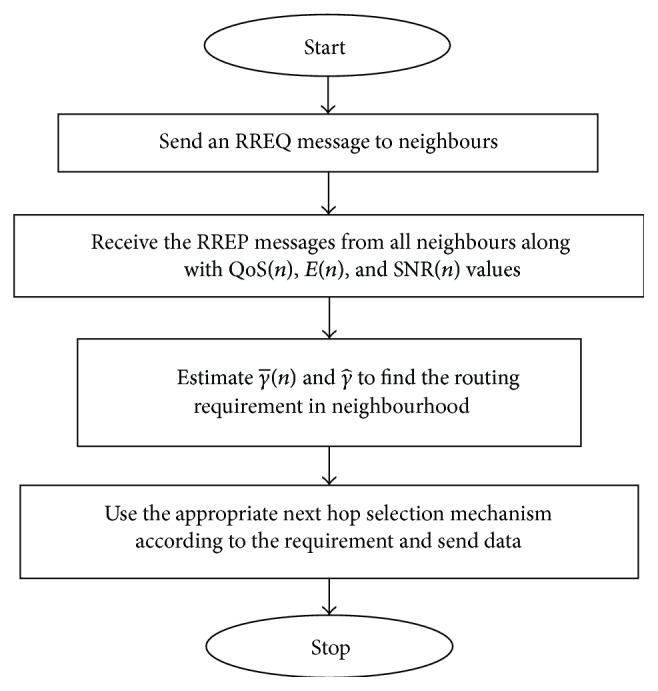
Working flow of APR.

The routing strategy does not use any tables unlike EDEAR. On-demand routing is known to be better for dynamic links and networks. Hence, a reactive routing mechanism is used for routing. The routing strategy is shown in Figure 5.

### 4.3. Limitations of Adaptive Priority Routing

APR does not provide enough evidence to be a reliable routing algorithm since it has many limitations. A node has to exchange information with all of its immediate neighbours to obtain the values of QoS, SNR, and residual energy, before transmitting data to next hop, which would consequently result in the growing ratio of message delivery, thus increasing the delay and energy consumption. Besides, it is possible that a node with low residual energy possesses high value of QoS or SNR, which can accelerate the consumption of its residual energy, thus making the routing reliability a serious threat in the network.

## 5. Quality Adaptive Priority Routing (QAPR)

**Figure 6:**
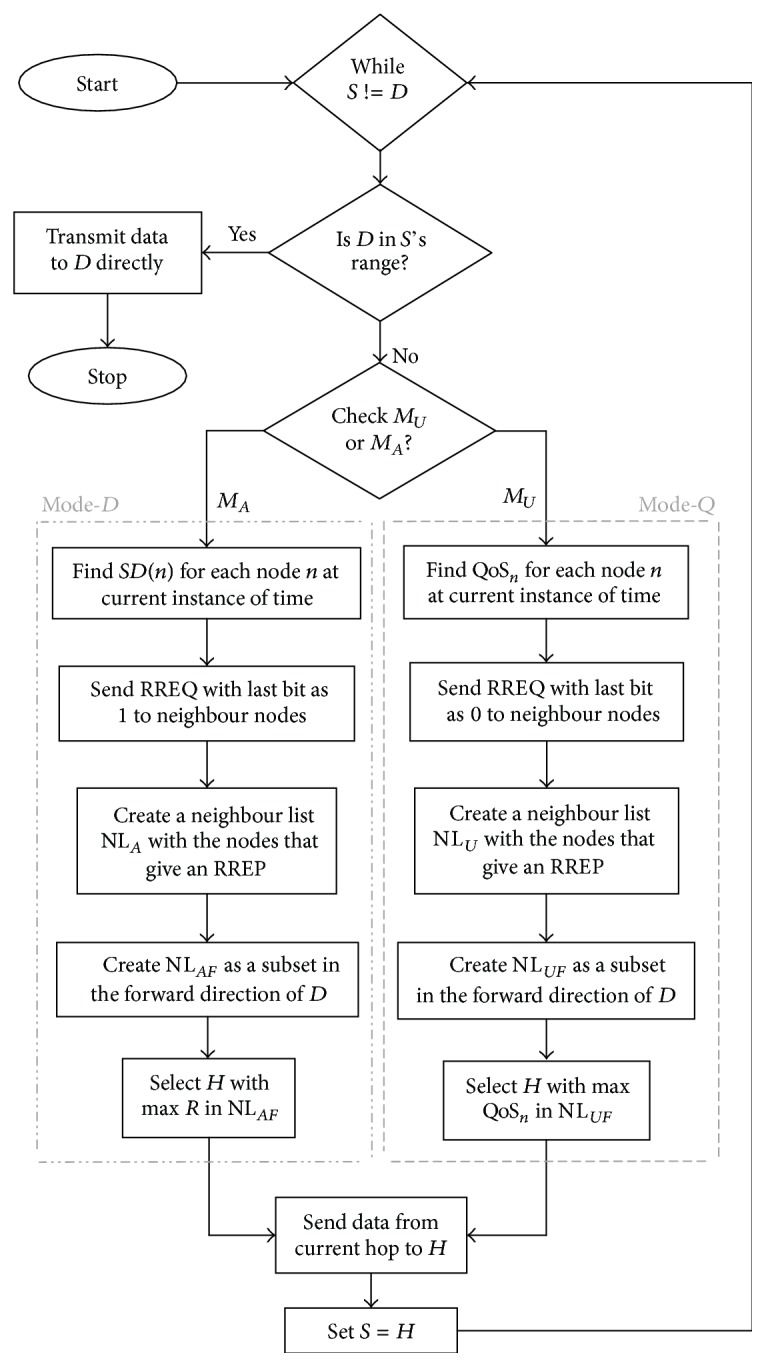
Working flow of QAPR-*D*.

To overcome the limitations of APR scheme, new Quality Adaptive Priority Routing (QAPR) is proposed. QAPR operates in two modes depending on whether the message packets sent are general message updates (*M*_*U*_) or alert messages (*M*_*A*_). The notation *D* represents the destination (base station/sink). Alert messages are sent with a much higher priority and the lowest possible delay, whereas the quality of service is a necessary metric for the normal update messages. The step-by-step process of QAPR is illustrated in Figure 6 using a flowchart showing both *Q* and *D* modes of operation.

Algorithm 1 corresponds to the basic and common operation of the QAPR protocol. If the sink *D* is in the direct range of the source* S*, then it sends data directly to destination.

### 5.1. Operation of QAPR in *D* Mode

**Figure 7:**
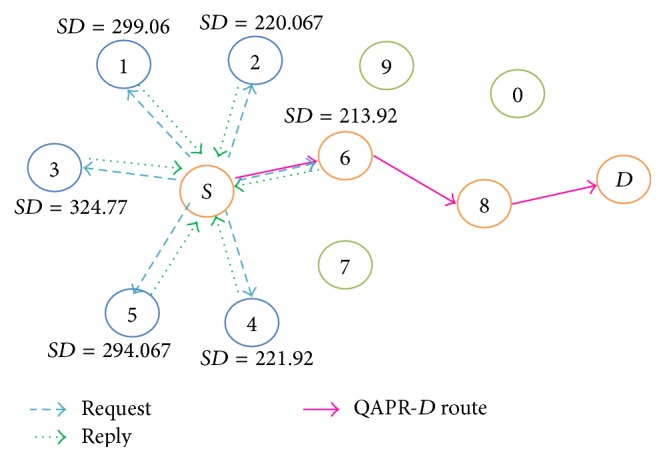
QAPR-*D* illustration with an example scenario.

In QAPR, distance from the sink and node reliability are taken as the priority while routing alert messages. Unlike APR that selects a next hop based on how far it is placed from the current node and a few other specific parameters, QAPR picks a node closer to the sink from a current node's neighbour list, whose strategy is explained with the illustration in Figure 7.

For all the nodes in the subset NL_AF_, a reliability factor *R* is estimated to find the most efficient next hop using

(11) $$\begin{array}{c} R=\frac{\text{No of RREP}}{\text{No of RREQ}},\;\left\{0\leq R\leq 1\right\}, \end{array}$$

where RREP means the route reply and RREQ refers to the route request messages of the node in NL_*U*_ at the time of communication. The dynamic estimation and next hop estimation process is iterated to generate consecutive routes to the destination.

Bearing in mind that the source *S* wants to send data to the sink *D*, the following steps are executed. The forwarding list NL_AF_ is a subset of NL_*A*_ containing only the nodes in the forward direction of *D*.* findNextHop_M*_*A*_ *()* function given in Algorithm 2 picks out the member *H* with the minimum distance (*SD*(*n*)) to the sink from the forwarding list NL_AF_ with the maximum reliability *R*. Therefore, *H* is selected as the next best hop for data transmission. The protocol reduces the hops encountered since distance to the sink is estimated first. Consequently, there is a possibility to reduce the energy consumption of the network. The reliability of data transmission is increased by the dynamic estimation of the value of *R* for every node.

### 5.2. Operation of QAPR in *Q* Mode

For normal update messages (*M*_*U*_), the operation of APR in the *Q* mode ensures the quality of service requirements is met while reporting data to the sink. By and large, QoS reflects the rate at which the communication is performed and high QoS is also preferred for reliable communication.

The forwarding neighbour list is obtained similar to that of QAPR-*D*, but the high QoS node is selected to find the next hop using the value of QoS as in (12). Similar to the algorithm of QAPR-*D*, the algorithm takes source *S*, sink *D*, and neighbour list NL_*U*_ as input parameters. Also, the algorithm checks whether the source node is equal to destination node or not. However, instead of SD(*n*) value, the QoS factor is measured for every node in the forwarding neighbour list NL_*UF*_ (which is a subset of NL_*U*_ taking only the nodes in the forward direction of the sink). Therefore, the node *H*, a return value of the function* findNextHop_M*_*U*_ *()*, is the efficient next hop selected by the QAPR-*Q* mode. The corresponding sequence of operations is shown in Algorithm 3.

The formula for finding QoS factor, (QoS_*k*_), is given by (12). The estimation is exhibited dynamically in a WSN, in order to improve reliability. Hence,

(12) QoSk=1k∑0kPkt deliveredkPkt lossk+Delayk+SNRk,0≤QoSk≤1,

where *k* is the node in the forwarding list NL_*UF*_, Pkt  delivered(*k*) are packets delivered to *k*, Pkt  loss(*k*) are packets dropped by *k*, and SNR(*k*) is the Signal-to-Noise Ratio of RREP received from *k*.

The estimation of QoS_*k*_ is repeated for every successive node selection till the data reaches the destination. The advantage of using QAPR-*Q* is that a single QoS factor can assure reliability when compared to the QAPR-*D* which uses two factors: shortest distance to sink and the reliability factor. This reduces the energy consumption of the nodes thus distributing the tasks instead of using only shortest path, but more reliable paths for high priority packets. This regulates the operation of a WSN in an atomic power station delivering updates and alerts about the state inside the power plant.

## 6. Performance Evaluation

The performance of QAPR is analyzed by using the Network Simulator (NS2). The tool is a discrete event time driven simulator which is used to mainly model the network protocols. The simulation of the proposed scheme has 38 nodes deployed in the simulation area 500 × 500 m, as in Table 1.

The performance of the proposed scheme is evaluated by two separate analyses. In the first part, we analyze the simulation of APR and QAPR-*Q* and QAPR-*D* in the flat topology. In the second part, the incorporation of these protocols in a hierarchical MANET is achieved to ensure safe and successful routing of data. The parameters used for assessment are packet delivery ratio, packet loss ratio, delay, and throughput.

### 6.1. Performance Metrics

The parameters used for assessment are packet delivery ratio, packet loss ratio, average delay, and throughput. The definition of each metric used to assess the performance and the corresponding formula is discussed here.

Packet delivery ratio (PDR) is the ratio of the total packets received to the total packets sent in a network. It is given by (13), where *h* represents the total number of nodes in the network {0 ≤ *h* < *∞*}:

(13) $$\begin{array}{c} \text{PDR}=\frac{\sum_{0}^{h}\text{Packets Received}}{\sum_{0}^{h}\text{Packets Sent}},\;\left\{0\leq \text{PDR}\leq 1\right\}. \end{array}$$

Packet loss ratio (PLR) is the ratio of the total packets lost in the network to the total number of packets sent. It is given by

(14) $$\begin{array}{c} \text{PLR}=\frac{\sum_{0}^{h}\text{Packets Lost}}{\sum_{0}^{h}\text{Packets Sent}},\;\left\{0\leq \text{PLR}\leq 1\right\}. \end{array}$$

The average time a node takes to process the information received is estimated as the average delay. It can be obtained as the average difference between the packet received time and packet sent time. It can be given by

(15) Avg.Delay=∑0hPktRecvTime−PktSentTimeh,0≤Avg.Delay<∞.

The total number of packets successfully received by all destinations in the network per unit time is measured as throughput here. It is estimated by

(16) Throughput=∑0hPktRecvTime ,0≤Throughput<∞.

Energy consumption is the total energy consumed per unit time per node in the network. Technically, it is the difference between the total initial energy per node and average residual energy of all nodes at the current instant of time. Residual energy (ResEnergy) is the amount of energy remaining in a node at the current instant of time. The energy consumption is measured using (17) and lies in the range {0 ≤ Avg.EnergyConsumption ≤ 1}:

(17) Avg.EnergyConsumption=InitialEnergy−∑0hResEnergyh,

where InitialEnergy denotes initial energy of a node in the network (1 joule) and ResEnergy denotes residual energy of the node *h*, {0 ≤ ResEnergy < *∞*}.

### 6.2. Analysis under Flat Topology

**Figure 8:**
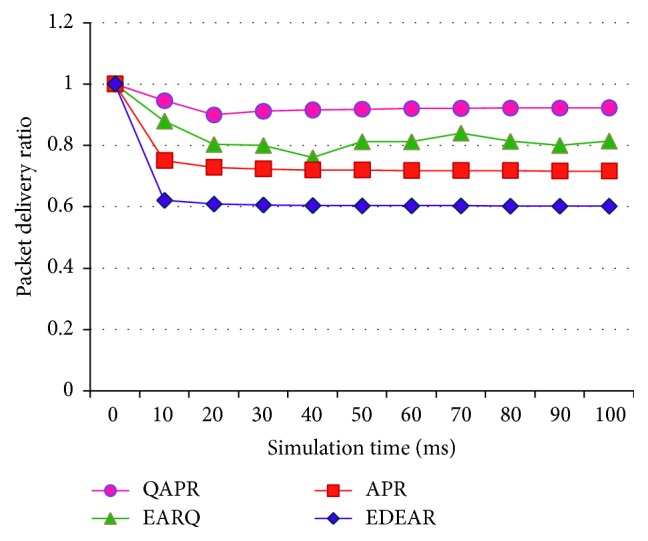
PDR of APR, QAPR, EDEAR, and EARQ under flat topology condition.

The working of APR, QAPR, EDEAR, and EARQ is assessed in the flat topology first in a 30-node scenario, during the development stage of the protocols. The flat topology analysis is more like an intermediate stage of the development of the proposed protocols. The packet delivery rates of APR, QAPR, EDEAR, and EARQ have been measured and plotted in Figure 8.

**Figure 9:**
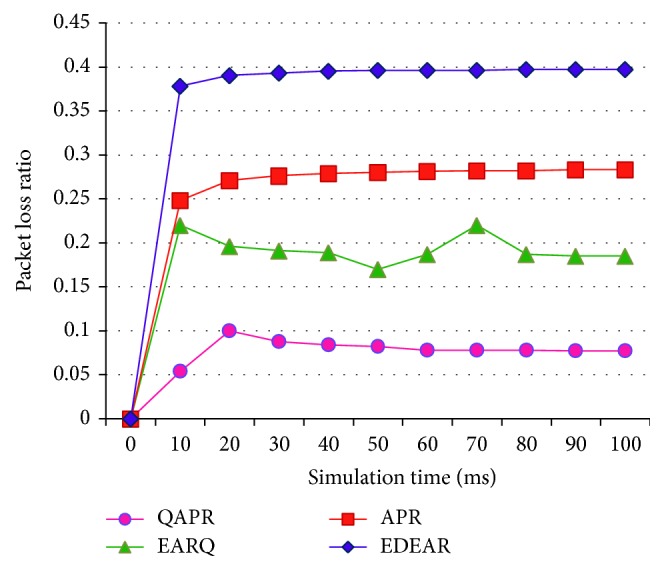
PLR of APR, QAPR, EDEAR, and EARQ under flat topology condition.

**Figure 10:**
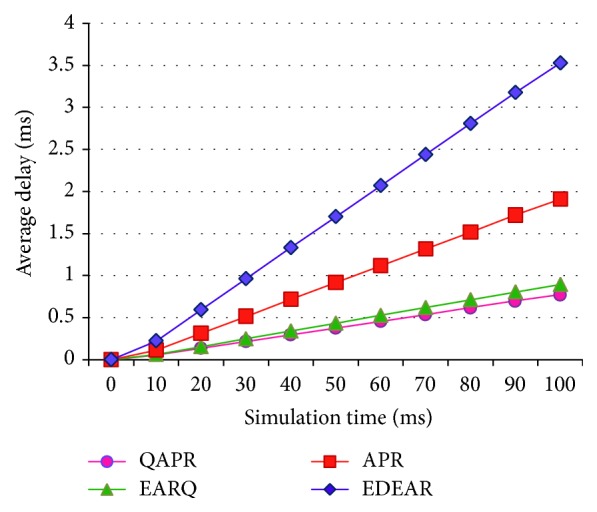
Average delays of APR, QAPR, EDEAR, and EARQ under flat topology condition.

The PDR of QAPR has the highest delivery rate when compared to all other protocols compared here. Being a proactive protocol, EARQ has the next highest delivery rate, which is about 11% less than the QAPR. APR shows 10% higher delivery rate than EDEAR protocol; however, QAPR outperforms EDEAR.  Figure 9 shows the plots obtained for APR, QAPR, EDEAR, and EARQ protocols. The PLR plot shows that QAPR-*Q* has the least amount of packet loss compared to the other protocols. The average delay per node of each protocol is obtained for all the four protocols APR, QAPR, EDEAR, and EARQ in Figure 10. This shows that both QAPR and EARQ have lesser amounts of delay compared to the other protocols. However, QAPR-*Q* shows 13% lesser amount of delay compared to EARQ although EARQ is proactive and QAPR is reactive.

**Figure 11:**
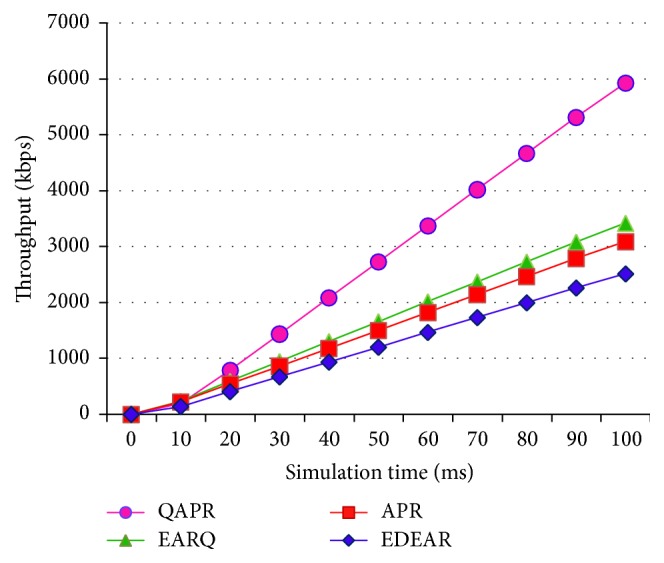
Throughputs of APR, QAPR, EDEAR, and EARQ under flat topology condition.

**Figure 12:**
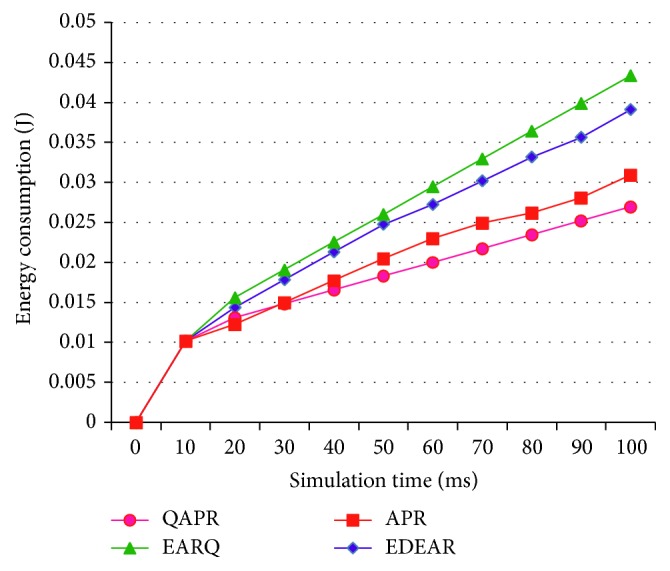
Energy consumption of APR, QAPR, EDEAR, and EARQ.

Throughput values of APR, QAPR, EDEAR, and EARQ are estimated and plotted in Figure 11. Clearly, QAPR performs better than all the other protocols. Since APR has different parameters measured at different hop selection periods, its performance is limited by the delay caused due to this. EARQ is proactive and therefore performs better compared to APR and EDEAR protocols. The initial energy has been set to 1 J per node and the number of transmissions and receptions impacts the energy of the node. The energy consumption per node is the average energy utilized by all the nodes. Energy consumed is measured and plotted for 100 ms network operation as shown in Figure 12.

The energy consumption is higher for EARQ when compared to the other three protocols proposed in this paper due to its proactive table updates. EDEAR is the second highest due to its parameter estimations while routing. APR reduces the number of hops while routing, therefore causing reduction in the network energy expenditure. QAPR is more specific in routing using a reliable node and hence the energy consumption is further reduced.

### 6.3. Analysis under Hierarchical Topology

Unequal clustering is performed for the various nodes in a network with the sink present beyond one of the edges of the rectangular area, with the deployment of the nodes totally randomly. Similar to the previous analysis, PDR, PLR, average delay, throughput, and energy consumption are shown for APR, QAPR, and REC+ since E-DEAR and EARQ do not support hierarchy.

**Figure 13:**
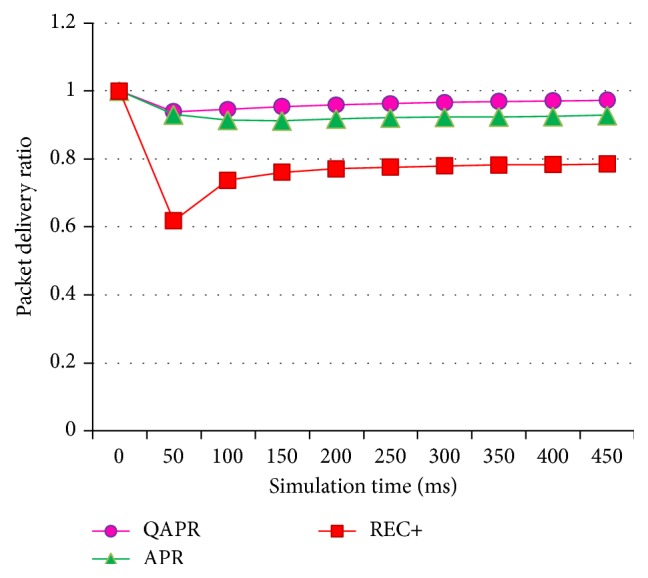
PDR of APR, QAPR, and REC+ under hierarchical topology condition.

**Figure 14:**
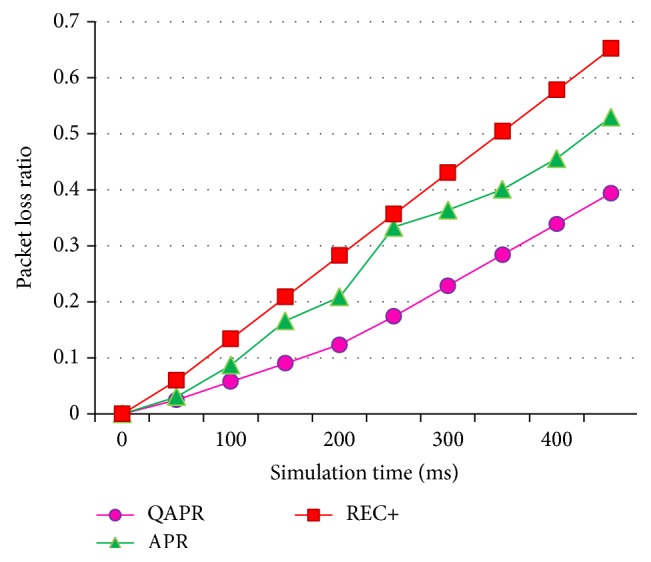
PLR of APR, QAPR, and REC+ under hierarchical topology condition.

Figure 13 shows the PDR for APR, QAPR, and REC+ obtained during the unequal clustering of a 50-node scenario. The PDR of QAPR is greater when compared to the PDR of REC+, which is in turn greater than APR, in a clustered WSN. Similarly, the PLR obtained during the simulation process is plotted in Figure 14.

**Figure 15:**
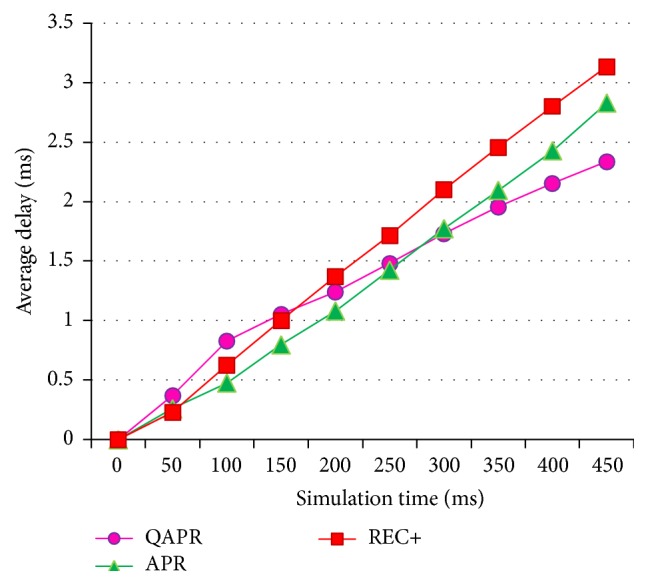
Average delays of APR, QAPR, and REC+ under hierarchical topology condition.

**Figure 16:**
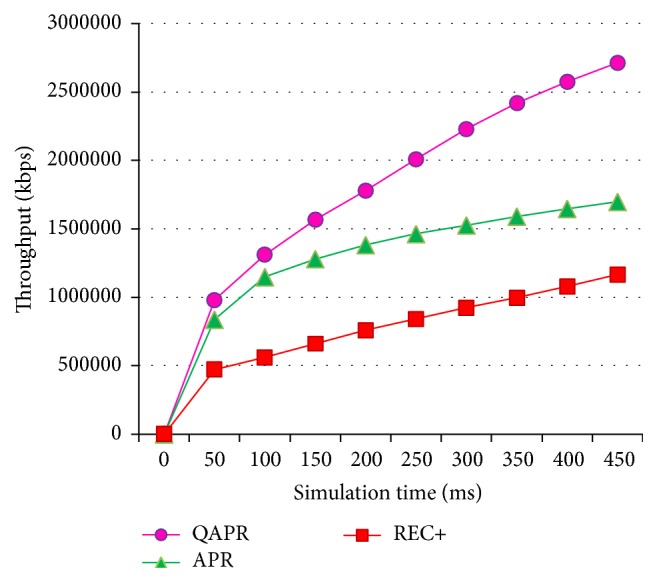
Throughput of APR, QAPR, and REC+ under hierarchical topology condition.

**Figure 17:**
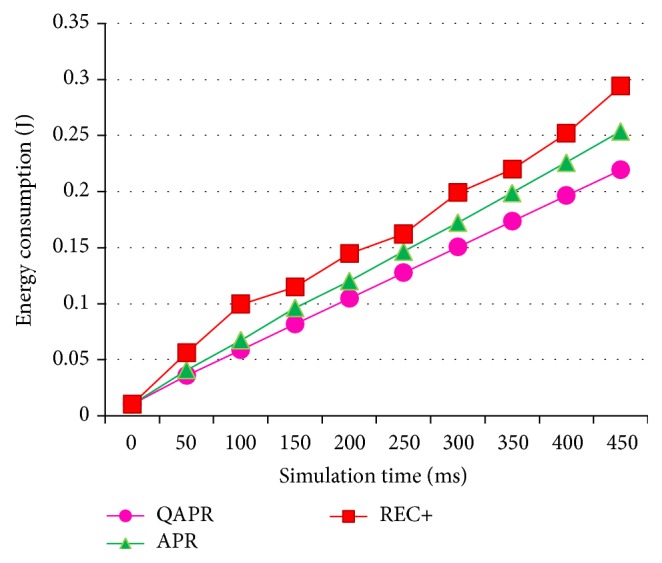
Energy consumption of APR, QAPR, and REC+ with hierarchical topology.

Since QAPR focuses mainly on the QoS factors, it performs better than both REC+ and APR protocols.  Figure 15 shows the average delays and Figure 16 shows the corresponding throughput. The energy consumption for APR, QAPR, and REC+ is plotted in Figure 17. The QAPR shows the lowest energy consumption making it preferable for energy-efficient and reliable routing compared to APR and REC+. QAPR and REC+ protocols perform 27.3% and 13.7% better than APR. Hence, for high reliability and critical applications like atomic power plant monitoring, QAPR is preferred. Since multipath routing induces extra 2 k energy consumption, multipath routing protocols are unlikely to be used in clustered multihop routing in WSN. Estimating hopwise reliability using QAPR and APR definitely increases the efficiency of routing in clustered multihop network. EARQ estimates the reliability of an entire path; however, the per-hop reliability estimation has provided greater reliability reflected in packet delivery, delay, and energy consumption metrics both in APR and further in QAPR.

## 7. Conclusion

This paper proposed an Adaptive Routing Protocol (APR) first to improve quality of routing in a WSN along with provision of single hop reliability. In other words, it helps in prioritizing over the type of next hop selection based on the requirement in the immediate neighbourhood among a reliable set of nodes. But a design limitation was identified and rectified to develop Quality Adaptive Reliable Routing (QAPR) to operate in two modes: QAPR-*D* and QAPR-*Q* for improved performance in the network providing high reliability and QoS. QAPR provides greater performance when compared to that of the APR, EDEAR, and EARQ protocols in the flat topology condition. A separate evaluation of the QAPR in the hierarchical topology condition of the WSN is performed against REC+ and APR. The analysis has proven that the multihop reliability provides lesser packet delivery than single hop reliability; that is, QAPR provides 27.3% lesser energy consumption than REC+. The single hop reliability is easy to estimate and provides guaranteed delivery for multihop clustered networks which makes QAPR suitable for atomic power plants requiring delivery of update and alert messages. Future works aim to provide security in multicast communication using distribution schemes [22].

## Figures and Tables

**Algorithm 1:**
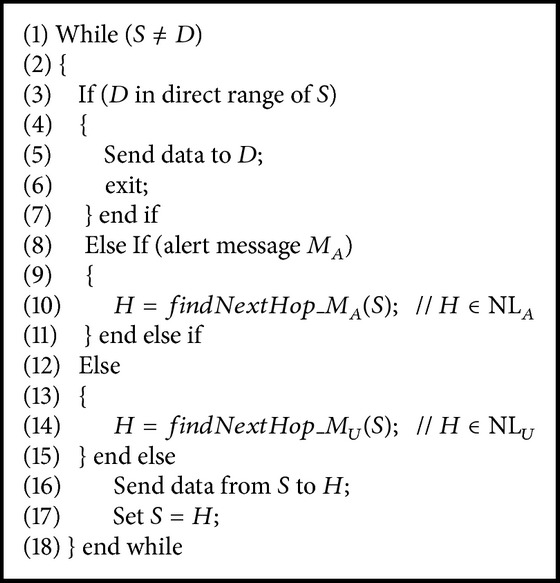


**Algorithm 2:**
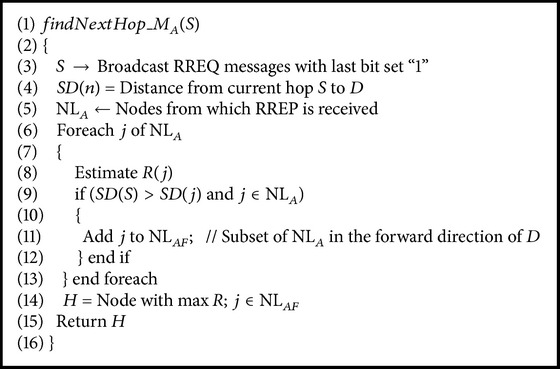


**Algorithm 3:**
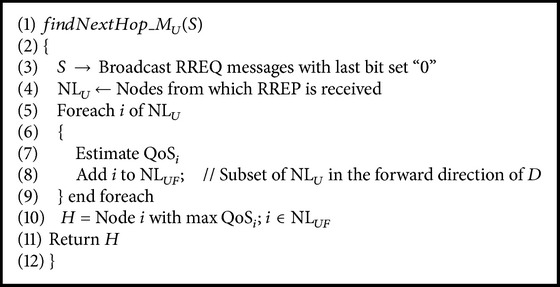


**Table 1:** Simulation parameters.

Parameter	Value
Simulation area	500 × 500 m
Channel type	Wireless channel
Network interface type	WirelessPhy
Antenna model	Omniantenna
Traffic model	CBR
Simulation time	100–500 ms
Number of nodes	Between 30 and 50
Initial energy	1 joule
